# Physical performance is enhanced in old mice fed a short term diet medicated with rapamycin, acarbose, and phenylbutyrate

**DOI:** 10.31491/apt.2021.03.051

**Published:** 2021-03-29

**Authors:** Megan Ellis, Warren Ladiges, Zhou Jiang

**Affiliations:** a Department of Comparative Medicine, School of Medicine, University of Washington, Seattle, WA 98195, USA

**Keywords:** Healthy aging, physical performance, aging processes, anti-aging drug cocktail, rapamycin, acarbose, phenylbutyrate, aging mice

## Abstract

Loss of physical performance, as seen in humans by decreased grip strength and overall physical fitness, is generally accepted to be a consequence of aging. Treatments to delay or reduce these changes or increase resilience to them are generally not available. In this preliminary study, 20-month-old male and female C57BL/6 mice were given either a standard mouse diet or a formulated mouse diet containing rapamycin (14 ppm), acarbose (1000 ppm), and phenylbutyrate (1000 ppm), or a diet containing one half dose of each drug, for 3 months. At the end of the study, performance on a rotarod and grip strength test was compared. In general, mice fed the full dose drug cocktail diet performed better on these assays, with significant improvements in rotarod performance in females fed the full dose cocktail and in grip strength in males fed the full dose cocktail, and females fed the low dose cocktail. These observations provide support for the concept that short term treatment with a cocktail of drugs that targets multiple aging pathways can increase resilience to aging, and suggests that this prototype cocktail could be part of a clinical therapeutic strategy for delaying age-related loss of physical performance in people.

Aging is a complex multifactorial process, meaning that multiple pathways need to be targeted to effectively prevent or slow aging [1]. A number of molecular targets are well known for influencing aging, but only a few have been successfully targeted with individual drugs. Three drugs, rapamycin (Rap), acrobose (Acb), and phenylbutyrate (Pba), were selected to test as a cocktail. The rationale for the drug cocktail was based on validated anti-aging effects of the individual drugs, each targeting different but overlapping processes of aging. Rap blocks mTOR, a protein shown to integrate signals from growth factors and nutrients to control protein synthesis. The anti-aging effect of downregulating mTOR was confirmed by the NIA Intervention Testing Program showing that rapamycin extended lifespan in mice [2]. Acb is a popular type 2 diabetes medication used for glucoregulatory control [3], and it also increases mouse lifespan [4]. Phenylbutyrate (Pba) is clinically approved as an ammonia scavenger for urea cycle disorders in children, and is also an inhibitor of histone deacetylase. In aging mice, it enhances physical and cognitive performance [5].

Male and female C57BL/6 mice were fed a diet containing Rap (14 ppm), Acb (1000 ppm), and Pba (1000 ppm), a diet containing a half-dose of each drug, or a non-medicated control diet, for 3 months starting at 20 months of age. Mice were then tested in a rotarod activity system and a grip strength apparatus for determining physiological performance. The rotarod apparatus (Rotamax 4/8, Columbus Instruments, Inc.) allowed mice to walk on a rotating rod with speed increased by 0.1 RPM/sec from 0 to 40 RPM over 5 minutes or until all mice were dislodged as determining by an infrared sensor. The time in seconds was recorded for three trails with half hour resting time in between each trail. The grip strength test in mice is similar to the hand grip test for people in that it assesses the ability to grip a device with the front paw (Liu *et al*., 2014; Whitehead *et al*., 2014). Mice were positioned horizontally with front paws gripping the bar (Columbus Instruments, Inc.) and pulled back by the tail slowly and steadily until they released their grip. The test was repeated 5 times and peak force was recorded and normalized to body weight.

Female C57B1/6 mice receiving the cocktail diet had significantly improved rotarod run times (Figure 1A). Male mice also had increased rotarod ran times, with *P* = 0.08 for the full dose group and *P* = 0.10 in the half dose group. While these are not statistically significant with an alpha of 0.05, they show a trend towards increased physical health that could reach significance with a longer treatment time or larger groups. Both male and female mice receiving the full dose cocktail diet showed a significantly increased grip strength versus mice on the control diet (Figure 1B). Female mice receiving the half dose cocktail diet also showed increased grip strength, with a *P*-value of 0.08. The lack of significance at an alpha of 0.05 in this group could be due to a wider variance in outcome values, but the average was still higher than in the control group. The amount of time animals were able to stay on a rotarod was dose- and gender-dependent, with improvements seen most significantly in female mice receiving the full dose. Grip strength, which correlates strongly with physical fitness tests performed on humans, was improved in mice seemingly independent of the dose administered and gender in mice fed the full dose cocktail. These observations suggest that a combination of Rap, Acb, and Pba for as little as three months can increase physical performance in aging mice, with beneficial effects in both genders. Further studies with a larger number of animals and more outcome parameters could further validate efficacy of this drug combination, and provide the rationale for testing efficacy in other mouse strains and as well as other species at older ages.

## Figures and Tables

**Figure 1.**
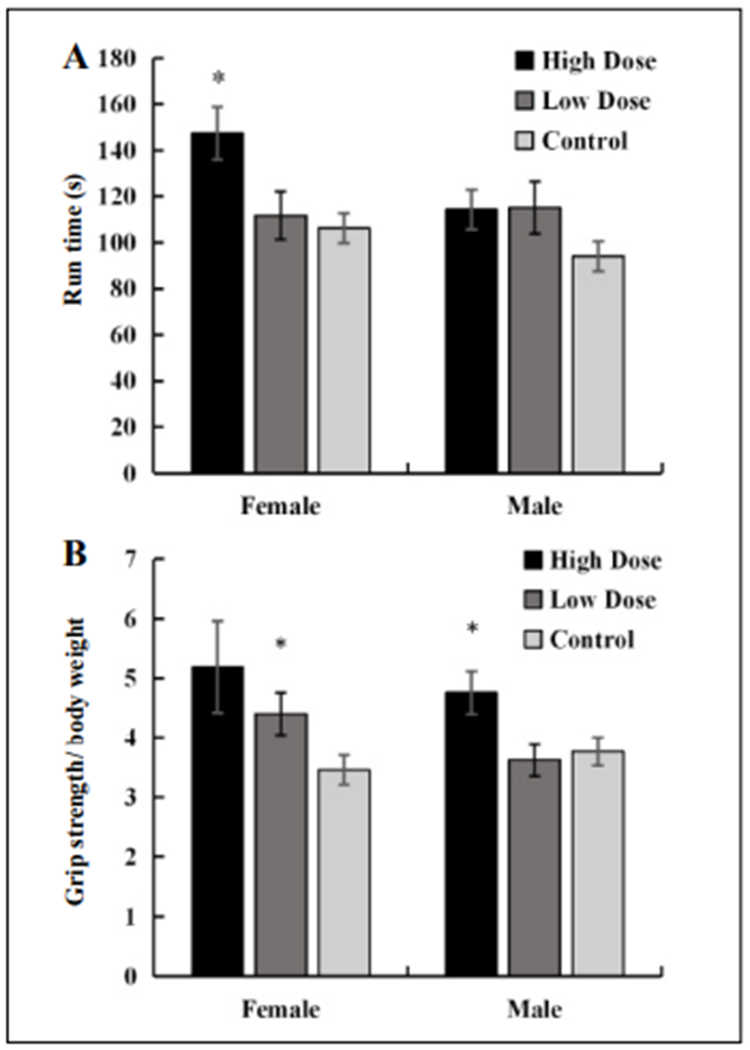
Female and male C57BL/6 mice, 20 months of age, were fed a rodent chow diet (high dose) containing Rap (14 ppm), Acb (1000 ppm) and Pba (1000 ppm), a diet containing one half the dose (low dose) of each drug, or a control nonmedicated rodent chow diet for three months and then tested for physical performance using a rotarod and grip strength normalized to body weight. **(A)** The full dose cocktail treatment improved rotarod times in females but not males. **(B)** The full dose and half dose cocktail diets improved grip strength in females, but males only showed improvement with the full dose cocktail. *N* = 10-12; *Significance at *P* < 0.05, using student’s *t*-test.
